# Equity analysis of resource distribution for the Popular Pharmacy Program

**DOI:** 10.11606/S1518-8787.2019053000731

**Published:** 2019-05-15

**Authors:** Maria Eduarda de Lima e Silva, Aléssio Tony Cavalcanti de Almeida, Ignácio Tavares de Araújo

**Affiliations:** I Universidade Federal do Rio Grande do Sul. Faculdade de Ciências Econômicas. Postgraduate Program in Applied Economics. Porto Alegre, RS, Brasil; II Universidade Federal da Paraíba. Center for Applied Social Sciences. Postgraduate Program in Economics. João Pessoa, PB, Brasil; III Universidade Federal da Paraíba. Center for Applied Social Sciences. Department of Economics. João Pessoa, PB, Brasil

**Keywords:** Pharmaceutical Services, economics, Health Services Needs and Demand, Equity in Resource Allocation, National Policy of Pharmaceutical Assistance, Assistência Farmacêutica, economia, Necessidades e Demandas de Serviços de Saúde, Equidade na Alocação de Recursos, Política Nacional de Assistência Farmacêutica

## Abstract

**OBJECTIVE:**

To analyze the regional allocation of the resources from the Brazilian Popular Pharmacy Program, taking into account the relative availability of the program and the potential needs of the region.

**METHODS:**

Data from the National Health Survey of the Annual Report of Social Information and the administrative database of the program were used to create a non-parametric indicator of coverage using multiple data envelopment analysis technique. This indicator considers the relative availability of the program, taking into account equal access to equal needs (equity based on regional needs). The analysis of this indicator shows if the regions that most need pharmaceutical assistance are those that receive more resources from the Brazilian Popular Pharmacy Program.

**RESULTS:**

The states belonging to the richest regions of the country, Southeast and South, present wider relative coverage of the Brazilian Popular Pharmacy Program compared to poorer localities. In addition, the inequalities observed between locations are better explained by inefficiency in the transfer of resources to the basic component of pharmaceutical care than by the Brazilian Popular Pharmacy Program itself. According to the model, a 43.76% increase in the transfer to the basic component of pharmaceutical care would be required in order to improve equity, whereas the increase required by the Brazilian Popular Pharmacy Program is equivalent to 22.71%.

**CONCLUSIONS:**

Although the Brazilian Popular Pharmacy Program seeks to reduce the socioeconomic inequalities observed in access to pharmaceutical care, which integrates health care services, regional disparities in access to medicine persist. These regional differences are attributed mostly to allocation failures and problems in managing the conventional pharmaceutical care cycle provided through SUS pharmacies.

## RESUMO

**OBJETIVO:**

Analisar a alocação regional dos recursos do Programa Farmácia Popular do Brasil, levando em conta a disponibilidade relativa do programa e as necessidades potenciais da região.

**MÉTODOS:**

Os dados da Pesquisa Nacional de Saúde, da Relação Anual de Informações Sociais e da base administrativa do programa foram usados para criar um indicador não paramétrico de cobertura a partir da técnica de análise envoltória de dados múltipla. Esse indicador considera a disponibilidade relativa do programa, considerando a equidade de acesso para necessidades idênticas (equidade baseada nas necessidades regionais). A análise desse indicador mostra se as regiões que mais necessitam de assistência farmacêutica são aquelas que recebem mais recursos do Programa Farmácia Popular do Brasil.

**RESULTADOS:**

Os estados pertencentes às regiões mais ricas do país, Sudeste e Sul, apresentam maior cobertura relativa do Programa Farmácia Popular do Brasil em relação às localidades mais pobres. Ademais, as desigualdades observadas entre os locais são melhor explicadas por ineficiência no repasse dos recursos para o componente básico da assistência farmacêutica do que pelo Programa Farmácia Popular do Brasil em si. Segundo o modelo, para melhorar a equidade, seria necessário um aumento de 43,76% nos repasses ao componente básico da assistência farmacêutica, enquanto o aumento requerido pelo Programa Farmácia Popular do Brasil equivale a 22,71%.

**CONCLUSÕES:**

Apesar de o Programa Farmácia Popular do Brasil buscar atenuar as desigualdades socioeconômicas observadas no acesso à assistência farmacêutica, que integra os serviços de atenção à saúde, persistem as disparidades regionais no acesso a medicamentos. Essas diferenças regionais são atribuídas em maior parte a falhas na alocação e problemas na gestão do ciclo de assistência farmacêutica convencional prestada por meio das farmácias do SUS.

## INTRODUCTION

Chronic Noncommunicable Diseases (NCDs) are the leading causes of death in Brazil. The high prevalence of hypertension (15.6% of adults or 31.3 million people), diabetes (4.7% of adults or 9.5 million people) and asthma (4.4% of adults or 6.4 million) corresponds to a significant number of early deaths and loss of quality of life due to disability. These require follow-up with high complexity health care services and continuous pharmacological treatment, which represent a significant economic impact for society and for the patients themselves, requiring coping policies^1–3^.

According to data from the Family Budget Survey (POF) 2008-2009, medicine expenses corresponds to the largest share of family health expenses, especially for the poorest families ^4^ ^,^ ^5^ . For them, this expense accounted for 66% of health expenses, whereas in the richest families it accounted for 29%. Thus, family expenses on medicine in Brazil are regressive. This context makes it necessary to implement pharmaceutical assistance policies (PA) that can ensure the population’s access to pharmacological treatment, especially the continuum. One of the main causes of discontinuation of this type of treatment is family budget constraints, which can aggravate the patient’s condition and increase the public health system’s expenses with the provision of medical and hospital services^6–8^.

Given the difficulties families have to afford medicine, the federal government instituted the Brazilian Popular Pharmacy Program (BPPP). The program aims to ensure low-cost access to drugs considered indispensable for aggravations of wide prevalence in the population, whose treatment has a significant impact on the family budget, seeking to promote the integrity of health care and welfare gains ^9^ . Following the suspension of Rede Própria (RP) in 2017, the program started to operate as *Aqui Tem Farmácia Popular* (ATFP), , also known as a network agreement (NA), which consists of a public-private partnership between the federal government and the private network of pharmacies and drugstores. It was implemented in 2006, with the objective of expanding the program’s spatial coverage by using the existing structure of pharmaceutical retail.

However, the growing demand for health services coexists with the budget constraint and, inevitably, with the proposal to provide everything to everyone runs into the problem of limiting public resources ^10^ . Given the incompatibility between demand and public service offer, the national health policy is guided by the principle of equity in the provision of services to the population. Considering the importance of expanding drug access in the country, we analyze the regional allocation of BPPP resources, taking into account their relative availability and the potential needs of each region.

## METHODS

A cross-sectional study was carried out to identify the allocation of public resources directed towards the pharmaceutical assistance system passed onto the Brazilian states and the Federal District, taking into account the concept of equity and using an extension of the Data Envelopment Analysis (DEA) model, called analysis Multiple Data Envelopment Analysis (MDEA). This study follows the definition of equity proposed by the National Health Policy for guiding the actions developed by the Unified Health System (SUS), according to which care for individuals should be provided according to their needs, offering more to those who need more, and less to those who require less. Thus, by considering different living and health conditions, it seeks to meet the needs of different social groups ^a^ .

The DEA technique is an instrument for determining the relative efficiency of a decision-making unit (DMU), based on the approximation of an efficiency frontier built according to the comparison of the resources employed and the results obtained in a productive process. In this way, one can observe the units that present the best technologies in a given sample set. The efficiency points are those located on that border, whereas the DMUs below them are classified as inefficient ^11^ .

The empirical approach of this work was conceived considering the principles and directives of the National Health Policy of which the BPPP is part of, whose objective is to promote the balance among the benefited entities. In this sense, this evaluation follows the Puig-Junoy methodology ^12^ , which applies the DEA model to construct an equity indicator that matches local demands (needs) with the total value of the resources available for the service offering, taking into account the development of a border that indicates the maximum allocation of public services available to locations with similar needs.

The equity index (EI) obtained measures the relative deficit in service coverage provided by the distance between the observed point and the equity limit, indicating the inequality in the supply of services between locations with an array of similar needs. The locations below the equity limit have a deficit relative to the amount provided by the BPPP. In turn, the observations on this frontier are called equitable points from the perspective of the regional needs addressed.

For the implementation of the DEA approach, the productive units considered were the 26 Brazilian states and the Federal District. Given the limited number of observations and the relatively large number of input and output variables, equity estimates were obtained according to the extension method MDEA ^13^ ^,^ ^14^ .

The MDEA was proposed to overcome the problem of sample dimensionality, when many DMUs are arbitrarily identified as equitable. By this approach, it is possible to correct potential classification errors in the DMUs, increasing the discriminatory power of the method ^15^ . The MDEA technique computes the EI by applying the DEA for all possible combinations of input-output subsets for each DMU, making the comparison between the federative units fairer. Based on the frequency distribution of the calculated EIs for each sample unit, the mean can be acquired and used to identify and order the level of regional equity for the provision of AF ^15^ .

In order to compose the vector for MDEA needs, data about the level of income, proportion of older people and prevalence of CNCDs (hypertension, diabetes and asthma) were collected. The variables income and older people were chosen because they characterize a social dimension measurement that considers the issue of free or subsidized access to medicine. The measure of negative income was applied, since the BPPP aims to reduce the impact of drug costs on the domestic budget, a way to achieve a better distribution of public resources, so that relatively more financially vulnerable states receive more resources than more developed sites. The variables of the service vector were the value transferred to the drugstores registered in the ATFP and the amount transferred to the basic component of pharmaceutical assistance (BCPA) ^b^ .

The reference year of the study was established according to data from the National Health Survey (PNS) conducted in 2013 over the epidemiological profile related to CNCDs and the habits and living conditions of the Brazilian population, as well as access to and use of health services. In addition, data from the administrative and financial base of the BPPP and the Strategic Management Support Room of the Ministry of Health (SAGE/MS) ^c^ were used; the information regarding the number of drugstores by state was obtained in the Annual Social Information Report (RAIS) ^d^ . Table 1 presents the set of variables selected to estimate the EI and the descriptive statistics of the services vector in millions and of needs in thousands, paired with the variable tax income.

**Table 1:** Presentation and descriptive statistics of the selected variables for estimating the equity index per Brazilian state, 2013.

Variable	Mean	Standard Deviation
Needs		

Hypertension (thousands of people)	1.2	1.6
Diabetics (thousands of people)	337.84	506.9
Asthmatics (thousands of people)	238.44	333.2
Elderly (thousands of people)	978.07	1.4
Negative income (tax)	0.5351	0.7119

Services		

BPPP value (millions of reais)	67.6	100.2
CBAF value (millions of reais)	38	44.33

BPPP: Brazilian Popular Pharmacy Program; BCPA: Basic Component of Pharmaceutical Assistance

According to Table 1 , in the year 2013, the average volume of direct transfers transferred by the state to pharmacies in the CR modality of the PFPB was approximately 67.5 million reais, compared to approximately 38 million transferred to the CBAF. Analysis of the target diseases in this program shows that hypertension accounts for the highest proportion of chronic disease cases, with over one million diagnoses recorded, followed by diabetes and asthma, accounting for more than 300 and 200 thousand cases on average, respectively. The standard deviation shows great variability in the data, indicating that among the states there are favorable situations that coexist with significant deficiencies.

As this study aims to obtain the best availability of services within the same set of needs, the model was product-oriented so that service allocation was adjusted to meet local deficiencies. For the model estimation, the hypothesis of variable returns to scale (DEA-RSV) was assumed, considering the significant differences of socioeconomic factors and size of the federative units.

## RESULTS

Table 2 presents the EI calculated according to the MDEA for the 26 Brazilian states and the Federal District. A significant regional segregation of coverage indices is observed, wherein states belonging to the richest regions, South and Southeast, have better equity indices than the states of the North, Northeast and Midwest regions. Only one unit, the state of São Paulo, remained on the equity limit. The states with the lowest coverage are concentrated in the Northeast region, with Piauí having the highest relative deficit. However, the intersection between the Piauí, Pernambuco, Sergipe, Alagoas, Maranhão and Ceará confidence intervals is evident, demonstrating a homogeneity in fundraising between them.

**Table 2:** Equity index, standard error and confidence interval, 2013.

Major regions/states	Equity Index	Standard error	CI95%
North	0.7837*	0.0352*	0.7147-0.8527*
Tocantins	0.6788	0.0281	0.6237–0.7338
Acre	0.7411	0.0407	0.6613–0.821
Amazonas	0.7512	0.0418	0.6692–0.8332
Pará	0.753	0.0326	0.6892–0.8169
Amapá	0.7587	0.0396	0.6811–0.8364
Rondônia	0.8348	0.0227	0.7903–0.8792
Roraima	0.968	0.0183	0.9322–1.0038
Northeast	0.7289*	0.0283*	0.6735–0.7843*
Piauí	0.6197	0.023	0.5747–0.6647
Pernambuco	0.657	0.0255	0.6069–0.7071
Sergipe	0.6808	0.0302	0.6216–0.7399
Alagoas	0.6969	0.0299	0.6383–0.7555
Maranhão	0.697	0.0376	0.6233–0.7706
Ceará	0.702	0.0295	0.6443–0.7598
Bahia	0.8142	0.0322	0.7511–0.8774
Rio Grande do Norte	0.8295	0.0184	0.7935-0.8654
Paraíba	0.8629	0.0206	0.8225–0.9032
Southeast	0.928*	0.0382*	0.8531–1.003*
Espírito Santo	0.8319	0.0225	0.7879–0.876
Rio de Janeiro	0.9024	0.0114	0.88–0.9248
Minas Gerais	0.9778	0.0072	0.9637–0.9918
São Paulo	1	0	1.00–1.00
South	0.9054*	0.0426*	0.7808–0.9479*
Santa Catarina	0.8733	0.0186	0.8368–0.9097
Paraná	0.9162	0.0149	0.887–0.9454
Rio Grande do Sul	0.9267	0.0151	0.8971–0.9563
Midwest	0.7889*	0.0512*	0.7046–0.9051*
Mato Grosso	0.7128	0.0263	0.6613–0.7643
Mato Grosso do Sul	0.7412	0.0237	0.6947–0.7876
Distrito Federal	0.812	0.0241	0.7649–0.8592
Goiás	0.8896	0.0175	0.8553–0.9239

* Average values by region.

Given the inequality in the transfer of financial resources to the BPPP and the BCPA, Table 3 presents a comparison between the effective amount paid to the federative units and the optimal amount that should be transferred, in millions, so that they can reach the border of equity, under a scenario without budget constraints.

**Table 3:** Effective and projected values (in millions of reais) for BPPP and BCPA, under a scenario without budget constraint, 2013.

Major regions/states	Effective value			Projected value		
	
	BPPP	BCPA	Total	BPPP	BCPA	Total
North	44.76*	86.11*	130.87*	141.51*	605.46*	746.97*
Tocantins	5.1	8.19	13.29	18.51	25.05	43.56
Acre	0.29	4.04	4.33	9.87	78.2	88.07
Amazonas	0.98	18.47	19.45	17	222.3	239.3
Pará	25.63	41.2	66.83	50.13	76.05	126.18
Amapá	0.11	3.49	3.6	9.75	172.77	182.52
Rondônia	11.05	8.3	19.35	24.62	17.64	42.26
Roraima	1.6	2.42	4.02	11.63	13.45	25.08
Northeast	247.36*	292.64*	540*	477.51*	569.19*	1,046.7*
Piauí	13.73	16.69	30.42	34.12	38.56	72.68
Pernambuco	49.24	47.32	96.56	94.52	88.73	183.25
Sergipe	6.68	11.02	17.7	21.6	30.88	52.48
Alagoas	10.82	17.64	28.46	28.24	41.37	69.61
Maranhão	12.64	34.21	46.85	37.77	89.54	127.31
Ceará	36.64	47.58	84.22	71.63	89.29	160.92
Bahia	51.3	77.59	128.89	89.21	129.96	219.17
Rio Grande do Norte	35	18.91	53.91	53.26	28.84	82.1
Paraíba	31.31	21.68	52.99	47.16	32.02	79.18
Southeast	961.16*	424.44*	1,385.6*	1,024.36*	450.09*	1,474.45*
Espírito Santo	52.5	18.66	71.16	77.58	28.45	106.03
Rio de Janeiro	219.59	84.35	303.94	248.63	96.15	344.78
Minas Gerais	291.85	104.77	396.62	300.93	108.83	409.76
São Paulo	397.22	216.66	613.88	397.22	216.66	613.88
South	426.96*	146.81*	573.77*	496.66*	174.92*	671.58*
Santa Catarina	75.05	32.93	107.98	96.82	42.94	139.76
Paraná	130.81	56.62	187.43	150.55	65.65	216.2
Rio Grande do @Sul	221.1	57.26	278.36	249.29	66.33	315.62
Midwest	144.1*	76*	220.1*	220.26*	125.6*	345.86*
Mato Grosso	15.8	17.09	32.89	34.37	34.78	69.15
Mato Grosso do Sul	16.43	13.25	29.68	33.82	26.16	59.98
Distrito Federal	24.03	13.49	37.52	42.35	23.71	66.06
Goiás	87.84	32.17	120.01	109.72	40.95	150.67

Total	1,824.34	1,026.00	2,850.00	2,360.30	1,925.26	4,285.56

BPPP: Brazilian Popular Pharmacy Program; BCPA: Basic Component of Pharmaceutical Assistance

* Total amount per region.

The amount effectively transferred to the SUS pharmaceutical assistance corresponds to just over half of the transfers made to the BPPP. According to estimates computed by the MDEA, the increase in the amount passed to the BCPA required in order for all states to reach the equity limit would be 46.71%, whereas the BPPP would require 22.71%. The most significant differences between the effective value and the projected value of the BPPP are observed between the states of Amapá, Acre, Amazonas, Roraima and Tocantins, which receive only 1.1%, 3%, 5.8%, 13.8% and 27.6% of the optimal value, respectively. With regard to the BCPA, the data show the under-financing of the SUS’s PA system, especially in the cases of Amapá, Acre, Amazonas, Roraima and Tocantins, which should have increased their resources by 98.9%, 97.1%, 94.2%, 86.2% and 72.6%, respectively, in order to reach optimal allocation.

However, the results do not imply an increase in resources allocated to the BPPP or the BCPA. Table 4 shows the increase required for the amount passed onto both pharmaceutical assistance systems, in millions, so that locations with relative coverage deficits can achieve optimal allocation by reallocating resources already available for the aforementioned programs between the federative units and the Federal District. Therefore, this table presents the values to be transferred to the BPPP and the BCPA for each location, according to the equity criterion, under a scenario with budget constraint. In this case two hypotheses were considered for evaluate this transfer.

**Table 4:** Equity coefficient and projected values (in millions of reais) for BPPP and BCPA, under a budget constraint scenario according to type of relationship between programs, 2013.

Major regions/states	Complementary				Substitution			
	
	Coef. BPPP	Coef. BCPA	BPPP value	BCPA value	Coef. BPPP	Coef. BCPA	BPPP value	BCPA value
North			109.38*	322.68*			94.11*	402.7*
Tocantins	0.008	0.013	14.31	13.35	0.004	0.006	12.31	16.66
Acre	0.004	0.041	7.63	41.68	0.002	0.018	6.57	52.01
Amazonas	0.007	0.116	13.14	118.47	0.004	0.052	11.3	147.86
Pará	0.021	0.04	38.75	40.53	0.012	0.018	33.34	50.58
Amapá	0.004	0.09	7.53	92.08	0.002	0.04	6.48	114.91
Rondônia	0.01	0.009	19.03	9.4	0.006	0.004	16.37	11.73
Roraima	0.005	0.007	8.99	7.17	0.003	0.003	7.74	8.95
Northeast			369.07*	303.35*			317.58*	378.59*
Piauí	0.015	0.02	26.37	20.55	0.008	0.009	22.7	25.65
Pernambuco	0.04	0.046	73.06	47.29	0.022	0.021	62.87	59.02
Sergipe	0.009	0.016	16.69	16.46	0.005	0.007	14.36	20.54
Alagoas	0.012	0.022	21.83	22.05	0.007	0.01	18.78	27.52
Maranhão	0.016	0.047	29.2	47.72	0.009	0.021	25.12	59.55
Ceará	0.03	0.046	55.36	47.58	0.017	0.021	47.64	59.39
Bahia	0.038	0.068	68.95	69.26	0.021	0.03	59.33	86.44
Rio Grande do Norte	0.023	0.015	41.16	15.37	0.012	0.007	35.42	19.18
Paraíba	0.02	0.017	36.45	17.07	0.011	0.008	31.36	21.3
Southeast			791.77*	239.87*			681.33*	299.37*
Espírito Santo	0.033	0.015	59.96	15.16	0.018	0.007	51.6	18.92
Rio de Janeiro	0.105	0.05	192.18	51.24	0.058	0.022	165.37	63.95
Minas Gerais	0.128	0.057	232.6	58	0.07	0.025	200.16	72.39
São Paulo	0.168	0.113	307.03	115.47	0.093	0.051	264.2	144.11
South			266.04*	80.75*			330.34*	116.34*
Santa Catarina	0.041	0.022	74.84	22.88	0.023	0.01	64.4	28.56
Paraná	0.064	0.034	116.36	34.99	0.035	0.015	100.13	43.66
Rio Grande do Sul	0.106	0.035	196.69	35.35	0.058	0.016	165.81	44.12
Midwest			170.25*	66.93*			146.49*	83.54*
Mato Grosso	0.015	0.018	26.57	18.53	0.008	0.008	22.86	23.13
Mato Grosso do Sul	0.014	0.014	26.14	13.94	0.008	0.006	22.49	17.4
Distrito Federal	0.018	0.012	32.74	12.64	0.01	0.006	28.17	15.77
Goiás	0.047	0.021	84.8	21.82	0.026	0.01	72.97	27.24

Total			1,706.51	1,013.58			1,569.85	1,280.54

BPPP: Brazilian Popular Pharmacy Program; BCPA: Basic Component of Pharmaceutical Assistance; Coef.: coefficient

* Total amount per region.

The first investigates the allocation of financial resources by considering the programs complementary, since the federal government needs to finance distinct ways of dispensing subsidized drugs to the population; thus, PA programs act as competitors and require specific budgets. The second considers the programs as mutually exclusive, as there is a superposition of the amount of medicine distributed by them. Hence, a single budget would be considered for both PA public policies.

According to the first hypothesis, it can be seen in Table 4 that the transfer directed to BPPP is higher than the amount directed to BCPA, with the exception of the North region. According to the second hypothesis, in which case the BPPP serves to supplement BCPA’s deficiencies, the budget directed to BCPA overcomes the transfer values for BPPP only at the North and Northeast regions. In the case of the Southeast, South and Midwest regions, the situation is similar to that described by the first hypothesis: the transfers directed to BPPP overcome the values for BCPA.

The results generally demonstrate the prioritization of resources for BPPP. It can be observed that the expansion of the program is associated to socioeconomical factors in such a way that macro-regions with higher income end up having higher coverage. This relationship can be simplified by the Figure with the dispersion diagram generated by the estimated regression between the relative coverage deficit (complement of the equity index) and the ratio of accredited pharmacies in the NA modality of BPPP. Among the states from the South and Southeast regions, there is a high degree of adherence by private establishments to the agreed area, whereas the states of the North and Northeast regions face a high relative coverage deficit. The Midwest on the other hand is characterized as an intermediate scenario.

## DISCUSSION

Based on the equity criterion, this study evaluated the performance of the BPPP in meeting the pharmacological needs of the population through a comparative analysis between states. It can be seen that the equity indicators reflect regional inequalities, wherein states located in the richest regions are more assisted by the BPPP than the states from the poorest regions, North and Northeast.

In view of the presented scenario, the factors that can explain these regional inequalities are socioeconomic aspects or differences in the capture of resources transferred to the BPPP and the BCPA. This may signal problems in the management of pharmaceutical care provided by pharmacies in the public health system.

Considering that the states with the best equity rates are found in the richest regions of the country ^16^ , it can be verified that the territorial expansion of the program responds to commercial factors, to the detriment of the equity criterion, one of the principles that guide national healthcare policy actions. This can be attributed to the socioeconomic characteristics of the regions, due to the absence of initial criteria to guide the implementation of units from the agreed area. In this way, the expansion and distribution of medicine occurs due to the voluntary decision of private establishments to participate in the program. According to this hypothesis, drugstores located in more developed regions with a higher potential audience for the diseases covered by the program are more likely to participate in it ^17^ .

The regions that meet these conditions are the South and Southeast, which present a higher proportion of older people, better income indicators and lower inequality, ranking better than other regions with regard to the main factors pointed out in the literature as constituting barriers to medicine access ^18^ . On the other hand, the North and Northeast regions have less potential for consumption, both because they have lower income available to be spent on medicine and non-medicine products, and because they are still at the beginning of the aging population process. Thus, the incidence of CNCDs, more common with advanced age, is less observed in these localities than in the South and Southeast of the country. The association between these factors represents a lower demand for products commercialized in the pharmaceutical sector, which discourages drugstores from joining the ATFP. Hence why the BCPA has a greater projection in the North and Northeast regions, since the market in these localities is less attractive to the pharmaceutical retail sector, with it being incumbent on SUS pharmacies to promote the access to medicine.

However, when observing the performance of the states in providing the PA system in both alternatives, the BPPP is evidently beneficial to the detriment of the SUS PA system, as can be verified in the literature on the subject. Specifically, the results denote the insufficiency of the BCPA resources as the main cause of inefficiency in North and Northeast locations, such as Amapá, Amazonas and Bahia. Compared to the other regions, the greatest adjustment occurs for the resources destined to the BPPP. Therefore, under-financing and a possible mismanagement of the resources allocated to the public PA system can be verified. This result provides empirical evidence and corroborates with the hypotheses constructed by Santos-Pinto et al. ^10^ The authors, observing the high demand of BPPP by SUS users, affirm that this migration process in the search for pharmacological treatment can signal failures and inefficiency in the performance of SUS managers and local professionals in the pharmaceutical assistance cycle. In such cases, the BPPP is not in accordance with its role as an alternative for access to medicine, but represents the main access route to pharmacotherapy, even for those who depend on the public provision of medicine ^19^ .

This situation indicates that the beneficiaries who should acquire the medicine for free buy it through direct disbursement. The data analyzed do not only refer to the purchase of drugs prescribed for the treatment of hypertension, diabetes and asthma, which are exempt from co-payment, but also include transfers for the treatment of osteoporosis, rhinitis, Parkinson’s and glaucoma, which still require co-payment. In extreme cases occur the highest income compromise possible, stimulating the discontinuation of the drug regimen, associated with the worsening of the patient’s health condition. Hence, in some cases, the requirement of the co-payment rate may represent a cost higher than the families’ ability to pay, making it impossible for them to be served by the program ^20^ ^,^ ^21^ .

Despite a high rate of adherence to the program by pharmacies located in the South and Southeast regions, the results show that the national average participation of pharmaceutical units is low: 43% of private pharmacies are ATFP-enabled, so only 10% of the drug market is covered by it ^22^ . Due to the characteristics of the NA, the program’s most expressive part, the low adherence of businessmen in the sector restricts their geographical insertion, which implies the persistence of barriers that limit access to medicine in the country. It is therefore necessary for the federal government to adopt measures that promote the sustainability of the program by stimulating the participation of private drugstores, in order to guarantee the integral treatment of CNCDs and, thus, promote the efficiency of the health care system in the country ^23^ . From the perspective of the entrepreneur, given the complex market structure of pharmaceutical retail ^24^ , adherence to the program may represent a dissemination and demand increase strategy, increasing the profitability of accredited establishments. Hence, incentives granted by the federal government to pharmacies in order to participate in the ATFP may have benefits for society, businesses and the government.

It can be concluded that there are still barriers to medicine access in the country, especially in the regions that are poor and dependent on the public health system. The unavailability of medication makes it impossible to effectively adhere to pharmacotherapy, bringing complications associated with diseases that are out of control and generating additional costs for the public health sector. Given that the decline in quality of life and well-being compromise productivity and human capital accumulation, the reaffirmation of public policies that promote the expansion of health care services, including pharmaceutical assistance, is a factor that drives socioeconomic development in the locations served.

Finally, as this study used cross-sectional data, the results presented should be interpreted with caution, taking into account possible noises ^25^ . However, these results can be instruments to subsidize the elaboration of health policies that favor the efficient allocation of resources, in order to comply with the principles and guidelines defined by the national health policy, aiming for the social well-being and sustainability of public health financing.

**Figure:**
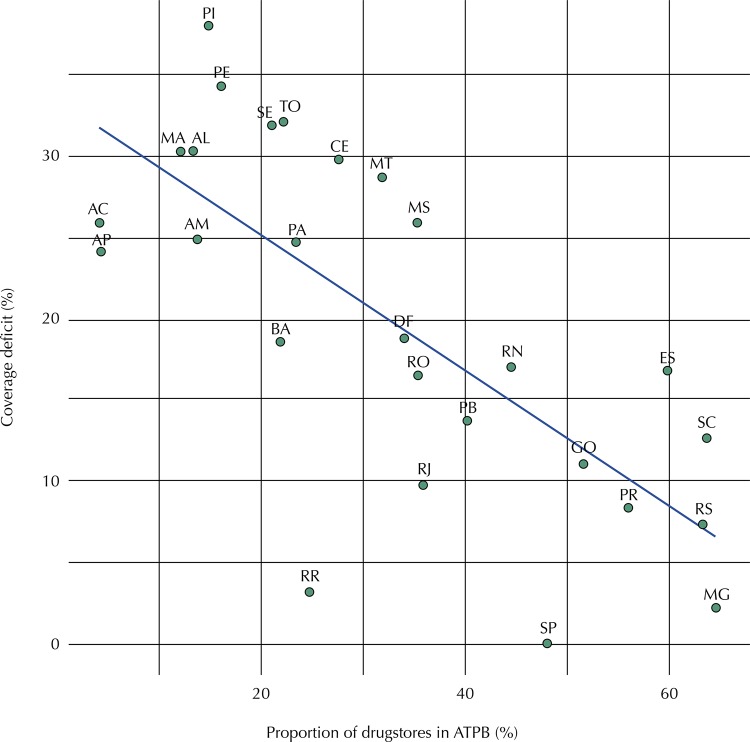
Dispersion diagram of the coverage deficit *versus* the proportion of drugstores registered in the program *Aqui Tem Farmácia Popular* (ATFP) per Brazilian state, 2013.

TO: Tocantins; AC: Acre; AM: Amazonas; PA: Pará; AP: Amapá; RO: Rondônia; RR: Roraima; PI: Piauí; PE: Pernambuco; SE: Sergipe; AL: Alagoas; MA: Maranhão; CE: Ceará; BA: Bahia; RN: Rio Grande do Norte; PB: Paraíba; ES: Espírito Santo; RJ: Rio de Janeiro; MG: Minas Gerais; SP: São Paulo; SC: Santa Catarina; PR: Paraná; RS: Rio Grande do Sul; MT: Mato Grosso; MS: Mato Grosso do Sul; DF: Distrito Federal; GO: Goiás

## Footnotes

a Fundação Oswaldo Cruz (BR). Pense SUS: equidade. Rio de Janeiro; s.d. [cited 2018 Jan 26]. Available from: https://pensesus.fiocruz.br/equidade

b The basic component of pharmaceutical assistance is related to the acquisition of medicine and supplies from the National Relation of Essential Medicine (RENAME), within the scope of Primary Health Care carried out by the Unified Health System (SUS). Ministério da Saúde (BR). Assistência Farmacêutica: Relação Nacional de Medicamentos Essenciais (Rename). Brasília, DF; s.d. [cited 2018 Jan 26]. Available from: http://portalms.saude.gov.br/assistencia-farmaceutica/medicamentos-rename/componente-basico-da-assistencia-farmaceutica-cbaf

c Ministério da Saúde (BR). Sala de Apoio à Gestão Estratégica [homepage]. Brasília, DF; s.d. [cited 2018 Jan 26]. Available from: http://sage.saude.gov.br/

d Ministério do Trabalho (BR). Relação Anual de Informações Sociais -RAIS. Brasília, DF; 2017 [cited 2017 Oct 10]. Available from: http://www.rais.gov.br/sitio/tabelas.jsf
